# Outsciencing the scientists: a cross-sectional mixed-methods investigation of public trust in scientists in seven European countries

**DOI:** 10.1136/bmjph-2023-000280

**Published:** 2023-12-12

**Authors:** Leonardo W Heyerdahl, Yanina Borzykh, Benedetta Lana, Anna-Maria Volkmann, Lars Crusefalk, Elien Colman, Nastassia Tvardik, Sibyl Anthierens, Muriel Vray, Tamara Giles-Vernick

**Affiliations:** 1 Global Health, Institut Pasteur/Université Paris Cité, Paris, France; 2 Anthropology, University College London, London, UK; 3 Sociology, Lund University, Lund, Sweden; 4 Department of Family Medicine and Population Health, University of Antwerp, Antwerp, Belgium

**Keywords:** Public Health, COVID-19, Epidemics

## Abstract

**Background:**

In this era of global health crises, public trust in scientists is a crucial determinant of adherence to public health recommendations. Studies of trust in scientists often link sociodemographic and other factors to such adherence but rely on assumptions about scientists and neglect scientific uncertainty. We undertook a cross-sectional mixed-methods study evaluating factors associated with public trust of scientists in Europe, investigating how and why respondents embraced certain claims in scientific debates.

**Methods:**

A survey was administered to 7000 participants across seven European countries in December 2020. Data concerning sociodemographic characteristics, trust in scientists, information source preferences, COVID-19 experiences and beliefs about pandemic origins were analysed using a multiple regression model. We employed thematic analysis to interpret open-text responses about pandemic origins and likely acceptance of treatments and vaccination.

**Results:**

Trust in scientists was associated with multiple sociodemographic characteristics, including higher age and educational levels, left/centre political affiliation and use of certain information sources. Respondents claiming that COVID-19 was deliberately released and that 5G technology worsened COVID-19 symptoms had lower levels of trust in scientists. Explaining their positions in debates about pandemic origins, respondents trusting and not trusting scientists invoked scientific results and practices, arguing that scientists were not the most important actors in these debates.

**Conclusions:**

Although our quantitative analyses align with prior studies, our qualitative analyses of scientists, their practices and perceived roles are more varied than prior research presumed. Further investigation of these variations is needed to strengthen scientific literacy and trust in scientists.

**WHAT IS ALREADY KNOWN ON THIS TOPIC**

- Available literature has demonstrated an association between the level of public trust in scientists and adherence to protective behaviours during epidemics and has emphasised the importance of effective public health communication to ensure compliance with public health guidance.

**WHAT THIS STUDY ADDS**

- The present study found that the distrusting public sought to ‘outscience’ the scientists, questioned who was a ‘scientist’, and contended that political and economic interests controlled scientific inquiry. Definitions of ‘scientists’ and ‘scientific investigation’ and perceived roles of scientists in epidemic emergence and policymaking are more varied than prior research presumed. Our study thus extends knowledge about trust in scientists by questioning assumptions about public definitions of ‘scientist’ and ‘scientific investigation’.

**HOW THIS STUDY MIGHT AFFECT RESEARCH, PRACTICE OR POLICY**

- This study expands the scope of research on trust in scientists by investigating qualitatively public understanding and definitions of scientists, scientific investigation and uncertainty. These insights should be integrated into strengthening scientific literacy in Europe.

## Introduction

 Preoccupations with public trust in scientists and science are not new.^1^ Longitudinal and multicountry surveys have assessed public confidence in science and scientists, in relation to sociodemographic factors such as age, gender, education level and political affiliation.^2 3^ Other studies have investigated specific factors affecting public trust in science and scientists, including perceived transparency, belief in pseudoscience,^4^ trust in government and corporations,^5^ conflicts of interest^6^ and historical cases of misuse of biomedical research.^7^ The COVID-19 pandemic accentuated public health specialists’ concerns about public trust in science and scientists,^8 9^ particularly because this trust is so closely linked to adherence to preventive measures, both non-pharmaceutical and vaccination.^10 11^ Past and current assessments of public trust in scientists and science have delineated similar profiles of social groups more or less likely to trust.^10^,^12^ Since 2020, they have also identified the global proliferation of controversies around pandemic origins, non-pharmaceutical interventions, vaccines and treatments.^13^ Facilitated by social media platforms and widespread anxiety in the recent pandemic crisis, certain social groups have been characterised as governed by emotion,^14^ irrationality,^15^ overconfidence^16^ and ignorance.^17^ Underpinning these studies addressing trust in scientists and science is an assumption that ‘scientists’ constitute a cohesive, homogeneous community engaged in a consensus-driven pursuit of knowledge.

Dissent, however, is at the core of scientific endeavour,^18^ and it is crucial for producing consensus, which is often fleeting.^19^ The profound uncertainties and debates characterising the scientific understanding of SARS-CoV-2, COVID-19, and prevention and treatment measures mirror the dissent that Thomas Kuhn found so fundamental to the production of scientific knowledge. In his seminal work ‘The Structure of Scientific Revolutions’, Kuhn argued that scientific progress is not always linear or cumulative. Instead, it often entails ‘paradigm shifts’, in which persistent anomalies and dissenting views challenge established frameworks of understanding, catalysing their replacement by new frameworks. The profound uncertainties and debates surrounding SARS-CoV-2, COVID-19, and its prevention and treatment measures provoked such Kuhnian debates and changing frameworks. Uncertainty during the pandemic also generated public health measures based on what Eysenbach^20^ has described as the ‘best available evidence’, and not ‘evidence-based facts’. Two salient questions remain unexplored in the literature on trust in science and scientists. First, proliferating surveys about public ‘trust in scientists’ and ‘trust in science’ without deeper exploration of lay understanding of who qualifies as a scientist and what scientists do misrepresent the complex and contentious processes of scientific knowledge production, particularly in uncertain and volatile epidemic contexts. We need better insight into the public’s understanding of what constitutes science, who can be considered a scientist and scientists’ roles—or lack thereof—in epidemic policymaking and response. Second, although numerous studies have examined levels of confidence towards science or scientists among different social and demographic groups, as well as associations between this trust and specific non-sociodemographic factors, there is scarce exploration and analysis of *why* the diverse public trust or distrust science and scientists.

We therefore conducted a cross-sectional mixed-methods survey among 7000 respondents in seven European countries (Belgium, France, Germany, Italy, Spain, Sweden and Ukraine) to investigate public trust in scientists engaged in COVID-19 research. Conducted in December 2020, the survey occurred at a crucial historical moment for public trust in scientists, when news of an effective COVID-19 vaccine had just been announced and plans for vaccine rollout were under way. The survey sought to identify diverse factors linked to the public trust in scientists involved in COVID-19 research, but also queried participants through closed-ended questions and text boxes about their understanding of pandemic origins and intentions to accept vaccination or specific treatments for COVID-19. Open-text responses revealed much about the public understanding of who are scientists, the work they do, the claims and practices they consider to be scientific, and the roles they and other actors have in producing knowledge to inform pandemic response.

## Methods

To develop the cross-sectional mixed-methods survey, we carried out social listening (online collection and analysis) of COVID-19-related tweets in English posted by users in the European Union in May–June 2020. We employed thematic coding to identify ongoing scientific controversies, top narratives circulating about scientists, trials, vaccines and treatments, including conspiracy-related discourses. We then used the results to create a survey investigating trust in scientists and in national and international authorities and institutions and included questions about the understanding of pandemic origins, and anticipated protective practices and devices, and treatment.

The cross-sectional survey, conducted by the market research firm Ipsos, was implemented through an online survey on 4–16 December 2020 among 7000 respondents in Belgium, France, Germany, Italy, Spain, Sweden and Ukraine. Following its standard protocol, Ipsos set quotas aligned with nationally representative proportions based on age (18–65 years), gender, geographical region and working status for each country. Ipsos developed a sample of participants from its existing online research panels, contacting potential participants by email to participate. When each quota was filled, Ipsos closed the quota immediately. One thousand respondents between 18 and 65 years old in each country participated. Ipsos did not survey those over age 65 years because its panel surveys cannot ensure representative sampling of this population. The following quantitative data were collected among respondents: socioeconomic and demographic characteristics, including gender identification (male/female/other/prefer not to answer); trust in sources of medical and scientific information; trust in national, European, and international institutions and authorities, as well as in pharmaceutical companies; perception of vaccine contents, purposes and safety; and political affiliation. The questionnaire included questions about participant understanding of clinical trials, perceptions of COVID-19 origins, prevention, testing, treatment preferences and anticipated COVID-19 vaccine acceptance. Trust in scientists was defined based on participants’ responses to three questions in the survey. Participants were asked to rank their level of agreement to the following statements:

1. ‘Scientists working in my country are competent to do research on COVID-19.’
2. ‘Scientists working in my country who are doing research on COVID-19 would be honest about what they discover.’
3. ‘Scientists working in my country who are doing research on COVID-19 are doing their work in the best interests of the public.’

### Data analysis

Data analyses were quantitative and qualitative. We initially conducted descriptive statistical analyses, presenting categorical variables as N (number of participants) and % (percentage from the total study population) for each category of variable. For analysis of trust, we combined the three questions above on trust into a single binary variable (trust/no trust), which served as a proxy to reflect trust in scientists. Relationships between trust in scientists and other factors (sociodemographic, information sources, personal COVID-19 experiences and beliefs in specific rumours) were analysed using an Akaike Information Criterion-based stepwise backwards multivariate regression model. We used France as the reference class. All results are expressed as ORs and 95% CIs. All quantitative analyses were performed using R software V.4.1.1.

Survey participants’ open-text responses about COVID-19 treatment, vaccination and SARS-CoV-2 origins were evaluated using thematic analysis.^21^ We developed a global codebook and conducted inductive and deductive thematic coding using NVivo software (QSR international, V.1.7.1). We also organised our analyses to categorise descriptions of existing COVID-19 scientific research according to expressed trust in scientists, participant sentiments towards key actors and explanations of responses contending that SARS-CoV-2 was deliberately released. Blank (unanswered) text boxes were not evaluated.

To ensure high-quality text analysis, a native speaker of each country language (French, Italian, Spanish, Ukrainian, German, Flemish and Swedish) performed the coding. All coders conferred frequently during the coding process to address and compare transversal codes and themes.

### Patient and public involvement

There were no patients involved in the study. We did not explicitly involve the public in the research questions, design, recruitment or outcome measures of the study, nor were they asked to assess the burden of time required to participate in the research. Our development of the survey tool, however, did draw on specific debates about COVID-19 on Twitter.

## Results

### Quantitative results

Our sample exhibited a balanced distribution of gender and age groups. In this sample, 34% participants were not employed, 43% identified as having centrist political beliefs and most possessed at least secondary education (see online supplemental file 1).

Table 1 evaluates trust in scientists and its associations with sociodemographic characteristics, use of information sources and beliefs, and experiences with COVID-19. Statistically significant associations were observed between trust in scientists and sociodemographic characteristics (country of origin, age, level of education, political affiliation). In comparison with France, respondents residing in Belgium (OR 1.25, 95% CI 1.03 to 1.52, p<0.025), Italy (OR 1.26, CI 1.04 to 1.54, p<0.020) and Sweden (OR 1.41, CI 1.16 to 1.72, p<0.001) had higher odds of trusting scientists. In contrast, respondents in Germany (OR 0.79, CI 0.65 to 0.96, p=0.018) and Ukraine (OR 0.39, CI 0.31 to 0.49, p<0.001) were less trustful of scientists. In comparison with the youngest respondents (18–24 years old), older groups, notably those 44–54 years old (OR 1.36, CI 1.12 to 1.65, p=0.002) and 55–65 years old (OR 1.71, CI 1.41 to 2.08, p<0.001), expressed significantly more trust in scientists. Participants with secondary (OR 1.33, CI 1.05 to 1.70, p=0.018) and tertiary (OR 1.57, CI 1.24 to 1.99, p<0.001) education levels also tended to trust scientists more than those with only primary education. Those declaring a preference to vote for centre (OR 1.15, CI 1.01 to 1.32, p=0.041) and left (OR 1.59, CI 1.33 to 1.89, p<0.001) political parties showed higher levels of trust than those affiliating themselves with politically right-wing parties.

**Table 1:** Relations between sociodemographic characteristics, information sources and beliefs, experience with COVID-19 and trust in scientists

	N	Crude			Adjusted		
		OR	95% CI	P value	OR	95% CI	P value
Sociodemographic characteristics							
Country of residence (vs France)	1000			<0.001			<0.001
Belgium	1000	1.16	0.97 to 1.38	0.11	1.25	1.03 to 1.52	**0.025**
Germany	1000	0.84	0.7 to 1.01	0.063	0.79	0.65 to 0.96	**0.018**
Italy	1000	1.24	1.04 to 1.48	**0.018**	1.26	1.04 to 1.54	**0.020**
Spain	1000	0.86	0.71 to 1.03	0.094	–	–	–
Sweden	1000	1.36	1.14 to 1.63	**<0.001**	1.41	1.16 to 1.72	**<0.001**
Ukraine	1000	0.32	0.25 to 0.39	**<0.001**	0.39	0.31 to 0.49	**<0.001**
Age (vs 18–24 years old)	916			<0.001			<0.001
25–34 years old	1455	1.16	0.96 to 1.39	0.12	–	–	–
35–44 years old	1546	1.27	1.06 to 1.51	**0.009**	–	–	–
45–54 years old	1640	1.54	1.29 to 1.83	**<0.001**	1.36	1.12 to 1.65	**0.002**
55–65 years old	1443	2.06	1.73 to 2.46	**<0.001**	1.71	1.41 to 2.08	**<0.001**
Gender (vs female)	3516			0.045			
Male	3478	1.13	1.03 to 1.25	**0.014**	–	–	–
Other	4	0.63	0.03 to 4.90	0.7	–	–	–
Education (vs primary or lower)	479			<0.001			<0.001
Secondary	3234	1.49	1.21 to 1.86	**<0.001**	1.33	1.05 to 1.70	**0.018**
Tertiary	3287	1.82	1.47 to 2.27	**<0.001**	1.57	1.24 to 1.99	**<0.001**
Working status (working vs not working)	4548 vs 2452	1.13	1.02 to 1.26	**0.018**	–	–	–
Marital status (single vs married)	2866 vs 4134	1.05	0.95 to 1.16	0.3	–	–	–
Political affiliation (vs right)	1282			<0.001			<0.001
Centre	969	1.12	0.98 to 1.27	0.086	1.15	1.01 to 1.32	**0.041**
Left	3025	1.62	1.38 to 1.91	**<0.001**	1.59	1.33 to 1.89	**<0.001**
Use of information sources (yes vs no)							
The media	5332 vs 1668	2.19	1.94 to 2.49	**<0.001**	1.48	1.28 to 1.72	**<0.001**
Internet websites (mainstream org)	2582 vs 4418	1.51	1.37 to 1.67	**<0.001**	1.39	1.24 to 1.55	**<0.001**
Blogs and non-mainstream websites	822 vs 6167	0.71	0.61 to 0.83	**<0.001**	0.81	0.68 to 0.97	**0.024**
Influencers on social networks	934 vs 6066	0.75	0.65 to 0.87	**<0.001**	–	–	–
Online written conversations with others	1516 vs 5484	0.80	0.71 to 0.90	**<0.001**	0.84	0.72 to 0.97	**0.016**
Face-to-face discussions with others	2367 vs 4633	1.24	1.12 to 1.37	**<0.001**	1.15	1.02 to 1.30	**0.021**
Articles shared on social media	2348 vs 4652	0.89	0.81 to 0.99	**0.035**	–	–	–
Healthcare professionals	1549 vs 5451	1.27	1.13 to 1.42	**<0.001**	–	–	–
Healthcare environment (eg, posters)	1188 vs 5812	1.27	1.12 to 1.45	**<0.001**	1.23	1.07 to 1.43	**0.005**
Other sources	596 vs 6404	0.99	0.83 to 1.18	>0.9	–	–	–
Have not found/received info from any	179 vs 6821	0.36	0.24 to 0.53	**<0.001**	0.62	0.40 to 0.94	**0.027**
Experience with COVID-19 (yes vs no)							
Admitted to hospital due to COVID-19	89 vs 6911	0.51	0.30 to 0.82	**0.008**	–	–	–
Had severe COVID-19	106 vs 6894	0.87	0.57 to 1.30	0.5	–	–	–
Tested positive for COVID-19	301 vs 6699	0.81	0.63 to 1.03	0.093	–	–	–
Had symptoms resembling COVID-19	771 vs 6229	0.92	0.79 to 1.08	0.3	–	–	–
Lost a family member to COVID-19	475 vs 6525	1.21	1.00 to 1.46	**0.055**	1.22	0.98 to 1.51	0.070
Close contact admitted to hospital for COVID-19	764 vs 6236	1.05	0.90 to 1.23	0.5	–	–	–
Close contact had severe COVID-19	769 vs 6231	0.99	0.85 to 1.16	0.9	–	–	–
Close contact tested positive for COVID-19	1955 vs 5045	1.31	1.17 to 1.45	<0.001	–	–	–
Close contact had COVID-19-like symptoms	1403 vs 5597	1.31	1.16 to 1.48	**<0.001**	1.18	1.03 to 1.35	**0.017**
No experience with COVID-19	3092 vs 3908	0.97	0.88 to 1.07	**0.5**	–	–	–
Experience with COVID-19 (ICU) (yes vs no)							
I have been critically ill with COVID-19 in ICU	296 vs 6704	1.00	0.78 to 1.27	>0.9	–	–	–
Close contact critically ill with COVID-19 in ICU	959 vs 6041	1.00	0.87 to 1.16	>0.9	–	–	–
Child critically ill with COVID-19 in ICU	155 vs 6845	0.59	0.40 to 0.84	**0.005**	0.72	0.48 to 1.08	0.12
Others critically ill with COVID-19 in ICU	1116 vs 5884	1.29	1.14 to 1.47	**<0.001**	1.21	1.01 to 1.44	**0.038**
No one clinically ill with COVID-19 in ICU	4161 vs 2839	1.16	1.05 to 1.28	**0.003**	1.24	1.08 to 1.42	**0.002**
Beliefs in controversial claims (yes vs no)							
Virus was deliberately released from lab	1364 vs 5636	0.34	0.29 to 0.39	**<0.001**	0.43	0.37 to 0.50	**<0.001**
COVID-19 symptoms caused by 5G tech	182 vs 6818	0.44	0.30 to 0.63	**<0.001**	–	–	–
COVID-19 symptoms worsen with 5G tech	253 vs 6747	0.36	0.26 to 0.50	**<0.001**	0.53	0.37 to 0.75	**<0.001**
Do not believe in any of above	641 vs 6359	1.02	0.86 to 1.20	0.8	–	–	–
COVID-19 vaccines contain microchips	981 vs 5016	0.49	0.42 to 0.57	**<0.001**	0.86	0.73 to 1.03	0.1

Bold values are statistically significant (p<0.05).

ICUintensive care unit

Use of certain information sources about COVID-19 was also significantly associated with trust in scientists. Participants obtaining their information via traditional media (newspapers, TV, radio, etc) (OR 1.48, CI 1.28 to 1.72, p<0.001), mainstream organisational/institutional websites (OR 1.39, CI 1.24 to 1.55, p<0.001), face-to-face discussions with friends and family (OR 1.15, CI 1.02 to 1.30, p=0.016) and their healthcare environment (eg, posters in hospital waiting rooms) (OR 1.23, CI 1.07 to 1.43, p=0.005) trusted in scientists more than those who did not. Respondents who reported seeking COVID-19-related information from blogs and non-mainstream websites (OR 0.81, CI 0.68 to 0.97, p=0.024), online conversations (OR 0.84, CI 0.72 to 0.97, p=0.016) and those who stated they did not use any information sources listed in the survey (OR 0.62, CI 0.40 to 0.94, p=0.027) were less trustful of scientists than those who did not.

Experience with COVID-19 was also associated with trust in scientists, including having a close family member or friend with COVID-19-like symptoms (OR 1.18, CI 1.03 to 1.35, p=0.017) or knowing someone who had been in an intensive care unit (ICU) due to COVID-19 (OR 1.21, CI 1.01 to 1.44, p=0.038). In addition, not knowing someone admitted to an ICU also yielded higher odds of trusting scientists (OR 1.24, CI 1.08 to 1.42, p=0.002).

Embracing narratives that significantly deviated from mainstream scientific debates about SARS-CoV-2 origins and purported roles of certain technologies in COVID-19 was significantly associated with decreased trust in scientists. Participants contending that COVID-19 was deliberately released from a laboratory (OR 0.43, CI 0.37 to 0.50, p<0.001) and that COVID-19 symptoms worsened with exposure to 5G technology (OR 0.53, CI 0,37 to 0.75, p<0.001) had much lower odds of trusting scientists than those indicating that they did not believe in these rumours.

**Figure 1:**
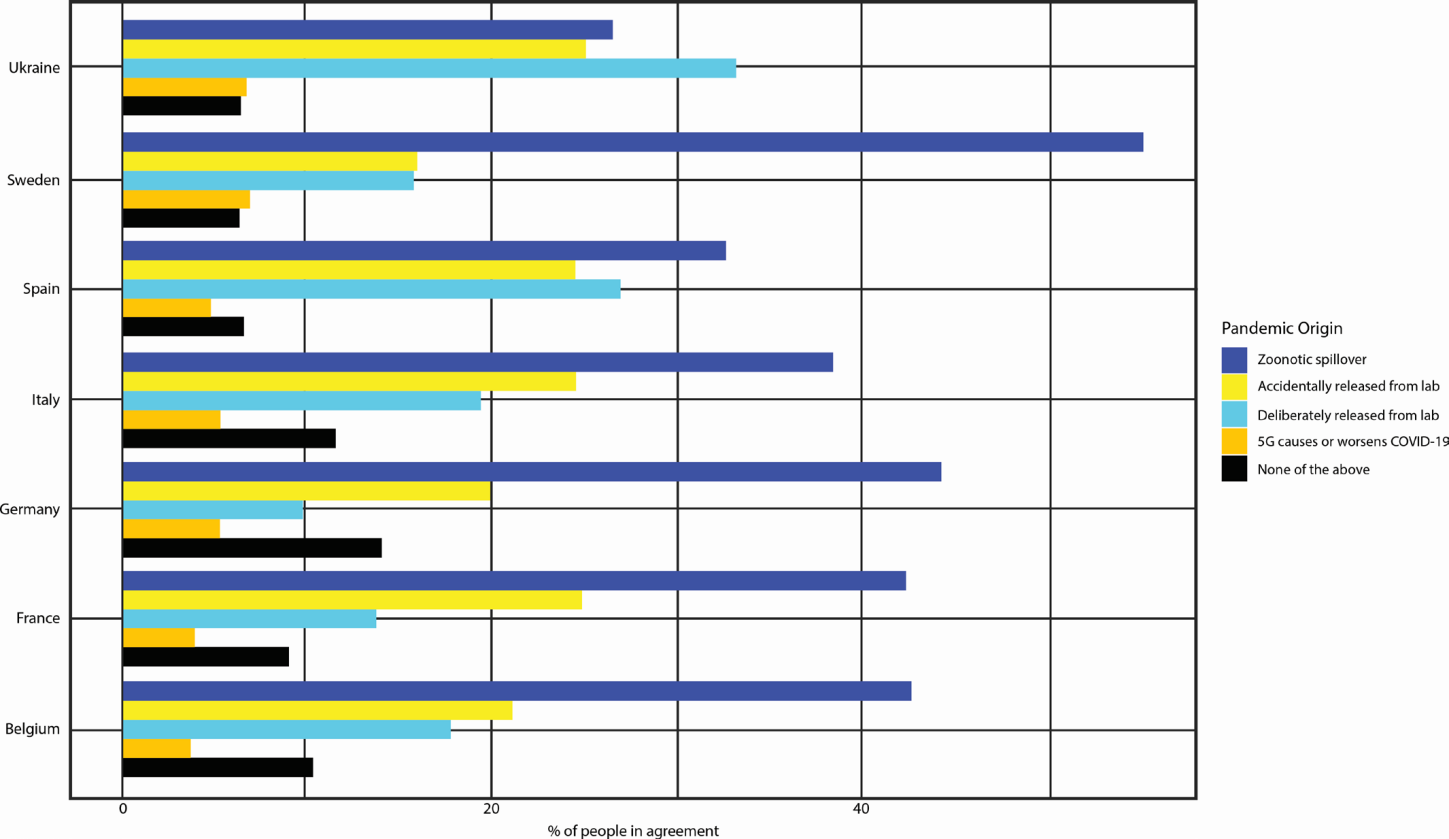
COVID-19 origins and aggravators by country.

The distribution of responses to COVID-19 origins and aggravators differed across countries, as shown in figure 1. In all countries except Ukraine, respondents most frequently attributed the pandemic origin to a zoonotic spillover. In Ukraine, however, respondents most frequently selected the response that the pandemic resulted from a deliberate viral release. Across all countries, at least one-third of respondents believed that the virus was accidentally or deliberately released. Claims that COVID-19 symptoms are caused or worsened by 5G technology were rare in all countries.

Table 2 shows that participants trusting scientists were more likely to believe that the COVID-19 pandemic origins resulted from a zoonotic spillover (OR 2.82, CI 2.55, 3.12, p<0.001) and to accept a COVID-19 vaccine (OR 3.67 OR, CI 3.29, 4.09, p<0.001). The relationship between trust in scientists and acceptance of paracetamol, the sole recommended treatment at the time, was not statistically significant (OR 1.1, CI 0.89, 1.35, p=0.4).

**Table 2:** Perceptions of COVID-19 origins and anticipated vaccine or paracetamol treatment acceptance among respondents trusting scientists

Characteristic	OR	95% CI	P value
Origins: zoonotic spillover	2.82	2.55, 3.12	<0.001
Vaccines: anticipated acceptance	3.67	3.29, 4.09	<0.001
Treatment: paracetamol	1.10	0.89, 1.35	0.4

### Qualitative results

Our qualitative results centred on text responses to queries about pandemic origins, intentions to accept specific COVID-19 treatments and anticipated COVID-19 vaccine acceptance. Overall, participants provided a total of 8404 open-text responses explaining their theories of pandemic origins (1859 responses) and intentions to accept a specific COVID-19 treatment (2205 responses) and a COVID-19 vaccine (4340 responses) (online supplemental file 2).

Roughly equal numbers of respondents trusting and not trusting scientists frequently referred to scientific research to justify their responses to questions about COVID-19 origins and intentions to accept COVID-19 vaccination and paracetamol as COVID-19 treatment (table 3). Participants also signalled a lack of data to support claims about treatments and vaccines. Those not trusting scientists tended to claim that existing knowledge was insufficient or that more research was needed. Crucially, these respondents mobilised apparently scientific justifications to support their contentions. One participant denying the pandemic’s cause was a zoonotic spillover argued, ‘No link established for animal transmission to date; ‘surprising’ viral sequence…’

**Table 3:** Trust in scientists and descriptions of existing COVID-19 scientific research

Qualitative codes	Participants distrusting scientists	Participants trusting scientists	Total
Cites existing research	201 (50%)	200 (50%)	401 (100%)
Requests additional research	147 (56%)	114 (44%)	261 (100%)
Claims lack of transparency in scientific results regarding COVID-19 origins, treatments or vaccines	72 (61%)	46 (39%)	118 (100%)
Claims studies were rushed	36 (58%)	26 (42%)	62 (100%)

Respondents not trusting scientists expressed roughly equally positive and negative comments about scientists (online supplemental file 3). Those not trusting scientists sometimes underscored scientists’ prowess to support their own convictions the virus was released intentionally. Still others lauded certain researchers who have taken stances against the dominant scientific discourse, notably Didier Raoult, who claimed that hydroxychloroquine was an effective COVID-19 treatment, and Franco Trinca, who advocated ‘free choice’ regarding COVID-19 vaccine uptake. Both Raoult (French dataset) and Trinca (Italian dataset) were applauded for their willingness to treat patients with drugs not recommended by national authorities (ie, hydroxychloroquine). Another respondent cited a ‘Nobel prize winner’ as a source for the claim that SARS-CoV-2 was not a ‘natural’ virus. Participants trusting scientists responded somewhat more positively about scientists but mentioned no individual scientists in their responses. Few scientists were named and applauded for their merits, but those mentioned had all challenged mainstream scientific discourses about COVID-19 origins and treatments.

Many respondents, although neither lauding or attacking scientists, suggested that scientists themselves were powerless, serving instead more powerful actors, including states, economic interests and pharmaceutical companies. Such reflections appeared in claims that scientists had developed COVID-19 vaccines long before the pandemic for authorities or had inserted microchips to control populations in these vaccines.

Even more revealing were open-text responses concerning pandemic origins, showing scientists as neither the most cited nor the most significant actors. Among participants contending that SARS-CoV-2 was deliberately released, we identified four themes in all country respondents (table 4). First, some respondents argued that the deliberate viral release was to impose demographic control, to reduce elderly and poor populations, and thus to decrease public spending on pensions, healthcare or social welfare. A second theme contended that the pandemic bolstered a country’s geopolitical standing. A French respondent, for instance, designated China as the cause of the pandemic, waging ‘a war without arms, aimed at weakening Europe and the U.S.’. China was the focus of much criticism (see also online supplemental file 3), with respondents contending that Chinese authorities had deliberately released the virus as part of its geopolitical strategy. Third, respondents argued the deliberate viral release was designed to reap financial benefits. A Ukrainian respondent contended that the pandemic was a means ‘to cause the final collapse in third world countries and further manipulate them for their own enrichment. Western European countries, the United States and China are enriching themselves.’ An Italian participant saw the beneficiaries as more circumscribed, arguing: ‘Behind every world catastrophe there is always a small circle of people … beyond nationality, ethnicity and government office…who make a huge profit at the expense of the general community’. Participants not trusting scientists were highly critical of pharmaceutical companies, arguing that these companies had deliberately released the pandemic virus to profit from vaccines. Finally, respondents contended that governments or ‘politicians’ released the virus to increase control over citizens. Another Italian participant observed, ‘The scoop is to further the Great Reset and create an Orwellian-style dictatorial world through the excuse of the pandemic’. A handful of participants offered other explanations, which were too diffuse or unclear to categorise.

**Table 4:** Frequency (Freq) of most common narratives in open-text responses about deliberate COVID-19 release

Narrative	Belgium (n=79)Freq	France (n=56)Freq	Germany (n=34)Freq	Italy (n=85)Freq	Spain (n=155)Freq	Sweden (n=58)Freq	Ukraine (n=127)Freq
Demographic control	+++	+++	+	++	+	++	+++
Geopolitical advantage	+	++	++	+++	++	+++	++
Financial profit	+	++	+	++	+	+	+
Social control	+	+	+	+	+	+	+
Other reason	++	+	++	+++	+	++	++
Unspecified	+++	+++	+++	++	+++	+++	+++

Noted+ for 0–9%, ++ for 10–19% and +++ for ≥20%.

Although these narratives appeared in all seven countries, their prevalence varied (see table 4). Open-text responses in Belgium, France and Ukraine highlighted demographic control as an explanation for the virus’s deliberate release, whereas Italy and Sweden tended to highlight its release as generating geopolitical advantages to other countries.

## Discussion

The present study investigated factors associated with European public trust in scientists at a crucial moment in the COVID-19 pandemic, exploring text responses concerning COVID-19 origins and intentions to accept COVID-19 treatments and vaccines. Our findings indicate higher levels of trust in scientists within higher-income countries of Belgium, Italy and Sweden, and less trust in Ukraine, a lower-income country. Consistent with previous research,^22 23^ we observed higher trust among older individuals, those in higher-income countries and those with higher educational levels. In contrast to a Wellcome Trust study,^22^ we found greater trust among respondents over 45 years old.

Political affiliation appears to play a role in trust levels. Our study suggests that those aligned with the political left and centre exhibited greater trust in scientists compared with their right-leaning counterparts, aligning with trends observed in the USA and Europe, where public perceptions of science have been influenced by political discourse.^24 25^ In contrast, one German study examining changing levels of trust in science over the pandemic reported that trust increased at its outset but declined over time, more so among right-wing voters.

Health information sources also influenced participants’ trust of scientists. Participants who used print and online newspapers, magazines, television, radio, news websites or apps, websites of mainstream organisations, as well as those who obtained information through personal conversations with friends and family or from healthcare environment trust scientists in their countries more than those who do not. Our findings differ from a broader literature on information sources during the COVID-19 pandemic, which have not addressed correlations with trust in scientists.^26^

Participants’ direct experience with COVID-19 (close contact with COVID-19-like symptoms or knowing someone who had been in an ICU), as well as not knowing someone admitted to an ICU for COVID-19, were positively associated with trust in scientists. These apparently contradictory findings, and particularly not knowing someone admitted to an ICU, could have resulted from the large sample size leading to more variables being statistically significant.

This study also found that beliefs in conspiracy-related narratives—that the pandemic resulted from a deliberate release of SARS-CoV-2 and that 5G technology exacerbated COVID-19 symptoms—were associated with lower levels of trust in scientists. Similarly, previous studies reported that belief in conspiracy theories was negatively associated with public trust in science^27^ and adoption of protective behaviours.^28^ The emergence and circulation of conspiracy narratives (beliefs that ‘major public events are secretly orchestrated by powerful and malevolent entities acting in concert’^26^) and an infodemic (a plethora of correct and incorrect health information) have been crucial features of this pandemic.^20 29 30^

Our analyses of open-text responses effectively recast our questions about public trust in scientists. First, we found that both respondents trusting and distrusting scientists supported their claims by citing existing scientific research and expressed a need for additional scientific data and research. These results suggest that respondents—even those not trusting national scientists—sought to employ evidence-based thinking. This finding appears to counter claims that those not trusting mainstream scientific discourse act out of emotion or irrationality.^15^ Although this question requires further investigation, our results suggest that scepticism of existing scientific knowledge on the eve of COVID-19 vaccine rollout in Europe fuelled demands for more scientific evidence. Whereas physicist Edwin Hubble^31^ argued that ‘a healthy dose of scepticism’ is a prerequisite to scientific thinking, European respondents in this study were highly sceptical of expert judgement. Although Atul Gawande indicates that a scientific mindset ‘observe[s] the world with an open mind, gathering facts and testing […] predictions and expectations against them’,^32^ distrustful participants in the present study signalled that they undertook similar approaches: they cited published works, they gathered observations, although anecdotal, but ones they considered to be facts. Appropriating this sceptical stance and scientific processes, respondents not trusting mainstream scientists across all surveyed countries suggests a desire to ‘outscience’ the scientists.

For Feinstein,^33^ laypeople are *outsiders* to science; they rely on scientific knowledge communicated to them. This outsider status uncovers a deep paradox of trust in science: the promise of modern science is to ‘know the truth instead of just trusting what you are told’, and yet trust in science is equally essential, when laypeople cannot surmount barriers of highly specialised, complex scientific knowledge.^34^ The information revolution once raised hopes for greater public participation in science,^19^ but new concerns about a post-truth era^35^ have displaced these earlier aspirations. Still, some respondent efforts to ‘outscience’ the scientists may reflect a continued desire to engage more fully in scientific investigation and findings, to discover truth for themselves. Respondents’ clamours for more data may also result from a conscious strategy to set impossible standards of certainty, to generate doubts and to postpone decision-making about viral origins, vaccines or treatments. Proctor and Schiebinger have described a similar strategy among interest groups engaged in scientific controversies.^36^

Second, our findings raised the question about who can be considered a scientist. Respondents who did not trust scientists in their own countries nevertheless appeared to mention and trust individuals whom they considered to be scientists, but who were controversial or whose status was disputed (eg, Raoult, Trinca). Some commentators have attributed lack of trust in science as the result of ‘fake experts […] who do not actually have a credible scientific track record’.^32^ Yet, individuals mentioned by respondents—even those roundly castigated by mainstream science for their records during the pandemic—remain difficult to dismiss as ‘fake experts’. Our results highlight the major challenges that the European public face in distinguishing ‘real’ from ‘fake’ scientists. A recent report, for instance, found that 65% of online anti-vaccine content originated with some 12 individuals; our further investigation into these individuals found that half declared that they possessed a medical or biomedical degree.^37^ The lay public may label active, influential critics as scientists, even when the latter disseminate inaccurate information in their online profiles, are banned from medical boards, or dismissed as pseudo-scientists in news outlets or peer-reviewed journals.

Third, we found that the participants’ rationale concerning pandemic origins or plans for COVID-19 treatment or vaccination did not attribute central roles to scientists. Certain respondents appeared to assign more pivotal roles to states, politicians and pharmaceutical companies, suggesting that an intentional viral release would enable powerful actors to reduce certain populations and expenditures on healthcare or social support, or to benefit economically from vaccines. Although these narratives were marked by the absence of scientists, they implied that scientists were nonetheless carrying out agendas of more powerful actors. These results align with Harambam’s findings^38^ that online Dutch conspiracy narratives often challenge the image of science as a collective, impartial search for knowledge, and that ‘science’, particularly biomedical research, is corrupted by the corporate world.

The four principal narratives identified in participants’ justifications for believing in the deliberate release of SARS-CoV-2—demographic control, geopolitical advantage, financial profit and social control—were observed across all countries, languages and cultural areas, although with varying intensities. That these narratives appeared across all country populations included in this survey suggest a shared cultural and linguistic ‘informational ecosystem’ across Europe.

Conspiracy narratives, despite their fallacies, offer a window into the underlying anxieties of those believing and spreading them.^39^ Demographic control narratives were especially prevalent in countries with ageing populations highly affected by COVID-19 in December 2020, notably France, Belgium and Italy. These countries are currently grappling with debates over funding their social retirement systems and sustaining their models of social support. The geopolitical advantage narrative, attributing the pandemic to a Chinese attempt to undermine the West, appeared to resonate with populations anxious about the emergence of a multipolar world, in which Europe and the USA no longer dominate as global economic, cultural and military powers. Respondents from all countries evoked this narrative, but more frequently in Italy and Sweden. Finally, the financial profit and social control narratives, possibly alimented by fears of concentrated power in the hands of private and/or state actors, were somewhat more prominent in France and Italy, which in recent years have been preoccupied by debates over accumulation of wealth and power by these actors.

All these narratives often align with and sometimes explicitly reference the Great Reset, a multifaceted conspiracy theory suggesting collusion between governments and large corporations to orchestrate the pandemic.^40^ Significantly, science and scientists do not feature prominently in such rationale. Scepticism or mistrust of science and scientists does not develop in a vacuum, but is produced and sustained by historical events that shape contemporary attitudes. Past abuses, such as the infamous Tuskegee Syphilis Study in the USA and the Mediator obesity drug scandal, demonstrate that unethical research practices and misapplications of scientific knowledge leave indelible traces on public memory, eroding long-term trust in science and scientists.^41^,^43^

One singular feature of our study is that it employed an approach that was initially qualitative, then quantitative, then mixed. We first conducted a thematic analysis of online discourse (social listening), using this analysis of the infodemic to inform our survey questions, which integrated key quantitative measures and open-text answers. Quantitatively, we evaluated trust indicators, gauged the prevalence of prominent pandemic conspiracy beliefs, and assessed anticipated vaccine acceptance and treatment preferences. Subsequently, our qualitative analysis highlighted fluid definitions of what constitutes a scientist; the common practice of citing sources to support claims about pandemic origins among both trusting and distrusting participants; four principal themes (demographic control, geopolitical positioning, financial benefit and political control over citizens) that underlay distrustful attitudes towards scientists consistent across nations; and an in-depth mapping of trust dynamics among actors mentioned in participants’ open rationales.

Mobilising and combining quantitative and qualitative methodologies leveraged the strengths of each approach. Upstream of the study, qualitative methods crucially highlighted previously unidentified variables through inductive characterisation of online discourse (social listening). These methods also contributed significantly to elucidating and analysing public explanations of their claims around pandemic origins and attitudes towards scientists. In turn, quantitative methods produced additional precision about key indicators of trust in scientists and associated factors and cross-country comparisons of predominant online discourses. Shuttling between and combining mixed-methods appear especially apt for analyses of rapidly changing, polarising and complex subjects like trust in scientists and science during an epidemic or pandemic.

### Limitations of the study

This study has multiple limitations. First, because the survey was administered online, respondents with better computer and internet access and higher levels of education were more likely to be recruited and to participate. Moreover, because Ipsos survey panels do not include participants over 65 years old due to uneven internet knowledge and use, we were unable to collect and analyse responses from older populations, which would have been illuminating.

Second, the survey was conducted over a 12-day period in December 2020, and in seven European countries. Our results are neither representative of all European countries, nor of high-income countries outside of Europe and middle-income and low-income countries (where populations may be less trustful of scientists). Trust in scientists may have changed significantly since December 2020. For this reason, our findings and conclusions are applicable to the seven European countries where the study was conducted. Although they reveal trust at a specific moment in the past, we employ these results to raise questions implicit in studies of trust in scientists and to encourage further investigation.

In investigating factors associated with trust in scientists, the present research did not address trust in science more generally. The survey contained specific questions about scientists’ honestly, integrity and intention to act in the interest of the public. ‘Science’ is a broad term, encompassing multiple actors, processes and practices. We compare our results with studies addressing both trust in ‘scientists’ and ‘science’, although we recognise that these terms do not have the same meaning for respondents.

The study’s large (N=7000) sample size in countries with diverse health, political, social, economic and cultural indicators, and conditions led to more statistically significant variables. A study of individual countries could shed additional light on more significant associations.

Finally, not all respondents explained their responses in text boxes. It is possible that respondents more adamant about their claims were more likely to respond to these specific questions. That said, in a previous publication, we found that responses to questions about intention to accept COVID-19 vaccination expressed conditionality.^44^ We would suggest, then, that these responses reflect a broad range of opinions, and not just those more convinced of their claims.

## Conclusion

This mixed-methods study of trust in scientists study integrated quantitative multivariate analysis and thematic analysis of participants’ open-text rationale. It produced statistically significant results on drivers of trust in scientists among the public in seven European countries, but also identified a shared reliance on evidence-based thinking among participants who trust scientists and those who do not, the relative erasure of scientists from participants’ rationale in favour of other actors and the predominance of controversial scientists among individual scientists.

These results should encourage additional investigation of trust in scientists beyond sociodemographic and other drivers, to explore public conceptions of scientists and of scientific investigation. They also should inform multipronged measures to enhance trust in scientists, which should include enhancing scientists’ visibility and emphasising their independence, as well as promoting greater public literacy about scientific investigation and uncertainty. Tackling the broader sociopolitical anxieties about public powerlessness in the face of powerful political and economic interests—which provide fodder for conspiracy narratives—may also indirectly strengthen trust in scientists.

Although our findings offer important insight into the dynamics of trust in scientists across selected European countries, they also underscore complexity of this trust. Given its crucial implications for public health policy and communication strategies, more granular investigations of the sociocultural, historical factors influencing public trust at national level are needed. Further research can guide more effective and nuanced science communication in the future.

## supplementary material

10.1136/bmjph-2023-000280 online supplemental file 1

10.1136/bmjph-2023-000280 online supplemental file 2

10.1136/bmjph-2023-000280 online supplemental file 3

## Data Availability

Data are available upon reasonable request.
