# Supplementary material for: Outsciencing the scientists: a cross-sectional mixed-methods investigation of public trust in scientists in seven European countries
Source: BMJ Public Health. 2023 Dec 12;1(1):e000280. doi: 10.1136/bmjph-2023-000280 (PMC11812721; doi:10.1136/bmjph-2023-000280)
Supplement: online supplemental file 1 [file bmjph-1-1-s001.pdf]

Supplement 1 Respondent characteristics, total and by country

Variable	TOTAL	Country						
		France	Belgium	Germany	Italy	Spain	Sweden	Ukraine
**Age**								
18-24	1000 (14%)	134 (14%)	132 (13%)	114 (11%)	110 (11%)	108 (11%)	157 (16%)	161 (16%)
25-34	1000 (14%)	200 (20%)	206 (21%)	192 (19%)	187 (19%)	212 (21%)	214 (21%)	244 (24%)
35-44	1000 (14%)	218 (22%)	215 (22%)	209 (21%)	258 (25%)	261 (26%)	180 (18%)	215 (22%)
45-54	1000 (14%)	224 (22%)	233 (23%)	259 (26%)	243 (24%)	229 (23%)	225 (22%)	227 (23%)
55-65	1000 (14%)	224 (22%)	214 (21%)	226 (23%)	212 (21%)	190 (19%)	224 (22%)	153 (15%)
**Gender**								
Female	3516 (50%)	511 (51%)	498 (50%)	497 (50%)	504 (50%)	498 (50%)	507 (51%)	501 (50%)
Male	3478 (50%)	487 (49%)	502 (50%)	499 (50%)	496 (50%)	502 (50%)	493 (49%)	499 (50%)
Other	4 (<0.1%)	1 (0.1%)	0 (0%)	3 (0.3%)	0 (0%)	0 (0%)	0 (0%)	0 (0%)
Prefer not to say	2 (<0.1%)	1 (0.1%)	0 (0%)	1 (0.1%)	0 (0%)	0 (0%)	0 (0%)	0 (0%)
**Work status**								
Not working	2452 (35%)	339 (34%)	359 (36%)	254 (25%)	432 (43%)	435 (44%)	244 (24%)	389 (39%)
**Education level**								
Primary or lower	479 (6.8%)	5 (0.5%)	126 (13%)	37 (3.7%)	81 (8.1%)	106 (11%)	72 (7.2%)	52 (5.2%)
Secondary	3234 (46%)	412 (41%)	350 (35%)	612 (61%)	669 (67%)	238 (24%)	549 (55%)	404 (40%)

Tertiary	3287 (47%)	583 (58%)	524 (52%)	351 (35%)	250 (25%)	656 (66%)	379 (38%)	544 (54%)
**Marital status**								
Married/Domestic partner	4134 (59%)	627 (63%)	552 (55%)	512 (51%)	574 (57%)	606 (61%)	601 (60%)	662 (66%)
**Political affiliation**								
Right	1282 (18%)	250 (25%)	243 (24%)	140 (14%)	232 (23%)	189 (19%)	339 (34%)	194 (19%)
Centre	3025 (43%)	421 (42%)	368 (37%)	606 (61%)	394 (39%)	458 (46%)	385 (38%)	393 (39%)
Left	969 (14%)	126 (13%)	111 (11%)	112 (11%)	170 (17%)	246 (25%)	141 (14%)	63 (6.3%)
NA	1724 (25%)	203 (20%)	278 (28%)	142 (14%)	204 (20%)	107 (11%)	135 (14%)	350 (35%)

All percentages were rounded up to whole numbers
