# Supplementary material for: Outsciencing the scientists: a cross-sectional mixed-methods investigation of public trust in scientists in seven European countries
Source: BMJ Public Health. 2023 Dec 12;1(1):e000280. doi: 10.1136/bmjph-2023-000280 (PMC11812721; doi:10.1136/bmjph-2023-000280)
Supplement: online supplemental file 2 [file bmjph-1-1-s002.pdf]

Supplement 2 Open text answers concerning COVID-19 origins and intentions to accept COVID-19 treatments and vaccine by country

Country	COVID-19 origin explanation	Treatment choice rationale	Vaccine acceptance/rejection rationale	Total
Belgium	262	351	631	1244
France	265	441	600	1306
Germany	164	224	644	1032
Italy	284	323	671	1278
Spain	379	323	646	1348
Sweden	206	278	626	1110
Ukraine	299	265	522	1086
Total	1859	2205	4340	8404
