# Supplementary material for: Outsciencing the scientists: a cross-sectional mixed-methods investigation of public trust in scientists in seven European countries
Source: BMJ Public Health. 2023 Dec 12;1(1):e000280. doi: 10.1136/bmjph-2023-000280 (PMC11812721; doi:10.1136/bmjph-2023-000280)
Supplement: online supplemental file 3 [file bmjph-1-1-s003.pdf]

**Supplement 3 Participant sentiments towards key actors, according to expressed trust or distrust in scientists**

| | Participants distrusting scientists | | | Participants trusting scientists | | |
|---|---|---|---|---|---|---|
| Identified actors | Negative sentiment | Positive sentiment | Total | Negative sentiment | Positive sentiment | Total |
| Pharmaceuticals | 67 (86%) | 11 (14%) | 78 (100%) | 19 (100%) | - | 19 (100%) |
| Health professionals | 2 (2%) | 94 (98%) | 96 (100%) | 3 (3%) | 107 (97%) | 110 (100%) |
| Politicians | 42 (74%) | 15 (26%) | 57 (100%) | 9 (45%) | 11 (55%) | 20 (100%) |
| Scientists | 65 (60%) | 44 (40%) | 101 (100%) | 32 (44%) | 41 (56%) | 73 (100%) |
| States | 160 (83%) | 32 (17%) | 192 (100%) | 94 (73%) | 35 (27%) | 129 (100%) |
| — China | 150 (86%) | 25 (14%) | 175 (100%) | 93 (82%) | 21 (18%) | 114 (100%) |
| — France | - | 6 (100%) | 6 (100%) | - | - | - |
| — Spain | 5 (50%) | 5 (50%) | 10 (100%) | - | 9 (100%) | 9 (100%) |
| — Sweden | 5 (71%) | 2 (29%) | 7 (100%) | 1 (17%) | 5 (83%) | 6 (100%) |
| — Ukraine | 3 (100%) | - | 3 (100%) | - | - | - |
| — USA | 31 (86%) | 5 (14%) | 36 (100%) | 10 (56%) | 8(44%) | 18 (100%) |
| Mass media | 24 (30%) | 56 (70%) | 80 (100%) | 8 (15%) | 47 (85%) | 55 (100%) |
| WHO | 5 (63%) | 3 (38%) | 8 (100%) | - | 1 (100%) | 1 (100%) |
| All actors | 273 (54%) | 234 (46%) | 507 (100%) | 124 (31%) | 277 (69%) | 401 (100%) |
